# Coronary computed tomographic angiographic imaging of cardiac allograft preserved in Paragonix SherpaPak Cardiac Transport System

**DOI:** 10.1016/j.jhlto.2024.100142

**Published:** 2024-08-03

**Authors:** Marian Urban, Brian D. Lowes, Stanley J. Radio, Ahmad Alshomrani, Marshall P. Hyden, Robbie Garvin, Kim F. Duncan, Nicholas W. Markin, John Y. Um, Chad Hovseth, Samer H. Sayyed

**Affiliations:** a Department of Surgery, Division of Cardiothoracic Surgery, University of Nebraska Medical Center, Omaha, Nebraska; b Department of Internal Medicine, Division of Cardiovascular Medicine, University of Nebraska Medical Center, Omaha, Nebraska; c Department of Pathology, Microbiology and Immunology, University of Nebraska Medical Center, Omaha, Nebraska; d Department of Anesthesiology, University of Nebraska Medical Center, Omaha, Nebraska

**Keywords:** heart transplantation, cardiac computed tomography angiography, donation after circulatory death, donor heart evaluation, Paragonix SherpaPak

## Abstract

Donation after circulatory death (DCD) heart transplantation is emerging as an alternative pathway to traditional donation after brain death (DBD) to expand the heart donor pool. Greater adoption of DCD heart allografts is hampered by the logistical and ethical constraints to perform invasive antemortem testing, thus limiting the capacity for the standard donor organ quality evaluation. Identification of the absence of coronary artery disease in patients at risk is an essential prerequisite for organ acceptance by an implant institution. This case presents a novel approach to the examination of coronary arteries in a cardiac allograft. We demonstrated that the coronary computed tomographic angiographic imaging of an ex-situ nonbeating cardiac allograft preserved after recovery in SherpaPak Cardiac Transport System is technically feasible. The images are of good quality and allow for three-dimensional reconstruction as well as quantification of coronary lesions.

## Background

Short- and mid-term outcomes of heart transplantation using cardiac allograft recovered from donation after circulatory death (DCD) have been shown to be noninferior to that after transplantation with a heart procured from donation after brain death (DBD).^1^, ^2^ One of the major barriers to greater acceptance of DCD cardiac allografts is the limited scope of pretransplant evaluation of potential donor’s organ quality. Evaluation of the coronary arteries in donors over 40 years of age, as well as younger donors at risk for coronary artery disease, is invariably required by transplant centers before acceptance of a heart for transplantation.^3^ For the brain-dead donor, coronary angiography is both routine and economically favorable.^4^ Organ donation after circulatory death is a regimented process that is independent from the compassionate end-of-life care. Invasive antemortem testing is often not performed due to ethical constraints. Many DCD hearts are therefore turned down due to insufficient allograft evaluation, including the unknown status of the coronary arteries. Identification of the absence of coronary artery disease after heart procurement would therefore greatly enhance the utilization of DCD cardiac allografts in donors at risk, for example, age ≥40, male gender, family history, smoking, hypertension, diabetes, hypercholesterolemia, etc.

Two different surgical techniques for the procurement of DCD hearts have been validated in clinical practice: direct procurement with ex-situ isolated heart normothermic machine perfusion and in-situ thoracoabdominal normothermic regional perfusion followed by either cold static storage or normothermic machine perfusion. Our team set to investigate the clinical utility of computed tomographic angiographic (CTA) imaging in evaluating the coronary arteries of a nonbeating cardiac allograft preserved after recovery in the Paragonix SherpaPak Cardiac Transport System (CTS).

## Case presentation

**Figure 1:**
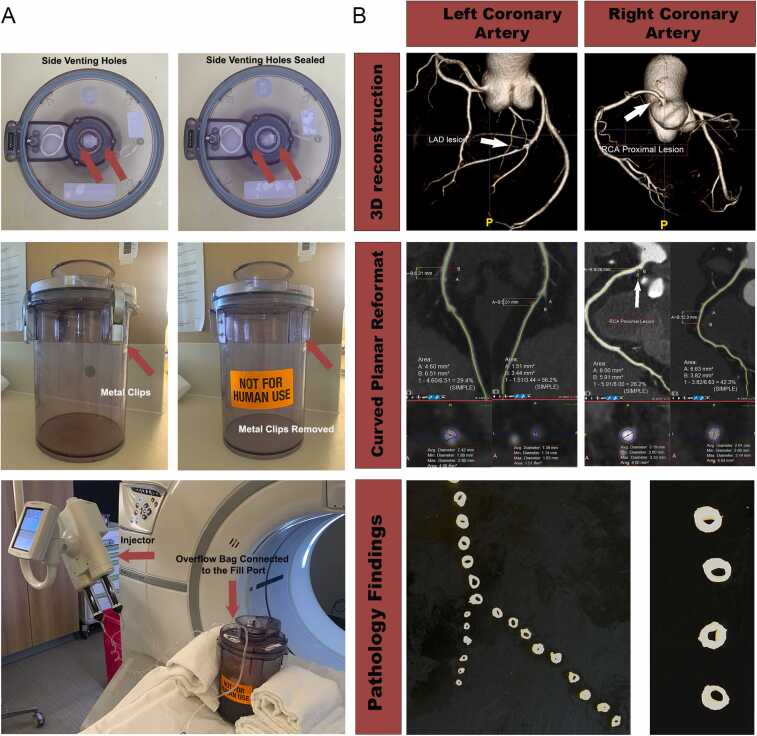
(A)Technical modifications and procedural details of coronary CTA of a donor heart in Paragonix SherpaPak. The side venting ports had to be sealed and metal clips removed from the commercially available device (red arrows). The contrast was injected through an extension tubing attached to the central vent port on the lid of the inner container. (B) 3D reconstruction of the aortic root and coronary arteries demonstrating lesions in the proximal RCA and mid LAD. Quantitative analysis was performed using curved planar reformat technique. Cross sections of the coronary arteries confirming luminal narrowing. 3D, three-dimensional; CTA, computed tomography angiography; LAD, left anterior descending; RCA, right coronary artery.

The donor was a 52-year-old male admitted with a gunshot wound to the head. Medical history included insulin-dependent diabetes and arterial hypertension. Despite decompression craniotomy, the patient failed to make progress and was transitioned to comfort care and planned withdrawal of life-sustaining therapies. After consent from the family was obtained, the patient was identified as a potential organ donor. The lungs, liver, and kidneys were allocated for transplantation; the heart was allocated for research. After direct procurement following the declaration of circulatory death, the heart was transported in the Paragonix SherpaPak CTS to our institution. Technical modifications of the commercially available device included the removal of the metal clips securing the lid to the inner container and sealing of the 2 side venting ports on the lid of the inner container (Figure 1A). Once inside the computed tomography (CT) scanner (GE Revolution 256 slice CT scanner), the central vent port communicating through the connector to the allograft’s ascending aorta was attached by the extension tubing to the Bayer MEDRAD Stellant FLEX CT Injection System. The fill port on the lid of the inner container was attached to a collection bag for the overflow fluid (Figure 1A). The total volume of 7 ml of iodine-based contrast (Isovue-370) and 13 ml of normal saline (35% contrast concentration) was injected at a rate of 2 ml per second. Images (0.625 mm slice thickness at 120 kVp and auto mA) were obtained with a 10-second delay from the injection to image acquisition. Radiation dose was noted at 70.69 dose lenght product (DLP). Coronary CTA was performed to assess the presence, location, severity, and composition of coronary plaques. Quantitative analysis was performed for all visually determined plaques using a dedicated software package (iNtuition TM version 4.6.0; TeraRecon) with additional three-dimensional rendering. CTA revealed a single, complex lesion in the mid left anterior descending (LAD) coronary artery (proximal calcification with 29% stenosis followed by a distal noncalcified (low attenuation) plaque with 56% stenosis). Proximal (26%) and mid (42%) right coronary artery (RCA) stenoses were also found (Figure 1B). Gross pathology examination noted normal coronary artery origins in a right dominant pattern. Pathology findings confirmed complex LAD lesion immediately after the takeoff of the septal branch with proximal calcified plaque followed by noncalcified soft plaque causing luminal narrowing estimated at 35%. There was also soft plaque present in mid RCA with 30% luminal narrowing. No acute thrombi were seen. (Figure 1B).

## Discussion

Previous reports demonstrated the feasibility of CT coronary angiography during ex-situ machine perfusion of explanted donor hearts in a beating state.^5^, ^6^ Our experiment demonstrated, for the first time, that the coronary CT angiography can also be performed on a nonbeating cardiac allograft cold stored in SherpaPak CTS. A limitation of cardiac CT is the image degradation due to cardiac motion. Imaging a heart at standstill eliminates this shortcoming and allows for three-dimensional reconstruction with quantification of coronary lesions. However, a drawback to the interpretation of the ex-situ CTA imagining is the lack of stimulated coronary vasodilation potentially resulting in an overestimation of disease severity. Fractional flow reserve—CT (HeartFlow) analysis to supplement anatomical data by assessing the hemodynamic significance of the lesion that is usually performed in-vivo is not feasible in an ex-situ setting.

CTA showed complex lesion in mid LAD and nonsignificant lesions in proximal and mid RCA. Pathology findings confirmed the presence of LAD lesion comprising of proximal calcific plaque followed by noncalcified lesion with significant cross-sectional luminal narrowing. As hypothesized, CTA was able to not only identify but also characterize the atherosclerotic lesions noted on gross pathology with high level of accuracy. Overestimation of stenosis severity between CTA and histopathology on postmortem specimens has been previously noted in the literature.^7^

Potential logistical challenges with CT coronary angiography of recovered cardiac allograft would involve timing of a recipient transplant. We estimate the time required to obtain the images and interpret the findings to be around 30 to 40 minutes. CTA done after allograft transport to the implant center would delay the commencement of the operation and potentially extend the cold ischemic time for the graft, especially in cases requiring complex recipient procedures. To overcome these limitations, CTA would need to be performed immediately after organ recovery at the donor center before being transported to recipient and have images pushed to recipient center for interpretation. This would inevitably add another layer of complexity to already very complex DCD heart recovery and could become a significant barrier to clinical adoption of this technique. For these reasons, we envision that CT coronary angiogram of cold-stored cardiac allograft will initially be utilized in cases with anticipated short cold ischemic times.

The ability to assess the coronary arteries of the heart allograft after procurement has the potential to significantly increase the donor pool of DCD allografts. Despite the tendency to overestimate the severity of luminal narrowing which may subsequently lead to discarding organs with nonhemodynamically significant lesions, the high negative predictive value of a normal CT coronary angiogram would ultimately lead to greater acceptance of DCD hearts from donors at risk for coronary artery disease.

## Disclosure statement

The authors declare that there is no conflict of interest that could be perceived as prejudicing the impartiality of the research reported.

Research reported in this manuscript was supported by Centers of Biomedical Research Excellence (COBRE) grant by National Institutes of Health (NIH) under award number P20 GM152326. All researchers are employed by Nebraska Medicine.

We recognize the contributions of LiveOn Nebraska staff and administration; the Nebraska Medicine clinical perfusion team (Lance Fristoe, Ryan Robertson, Todd Stover, Peyton Price, Jordanna Knott), and Jacob Dahlke (clinical ethicist).
